# Diversity in a Polymicrobial Community Revealed by Analysis of Viromes, Endolysins and CRISPR Spacers

**DOI:** 10.1371/journal.pone.0160574

**Published:** 2016-09-09

**Authors:** Michelle Davison, Todd J. Treangen, Sergey Koren, Mihai Pop, Devaki Bhaya

**Affiliations:** 1 Carnegie Institution for Science, Department of Plant Biology, Stanford, CA, 94305, United States of America; 2 Center for Bioinformatics and Computational Biology, Biomolecular Sciences Building, College Park, MD, 20742, United States of America; 3 Stanford University, Department of Biology, Stanford, CA, 94305, United States of America; 4 Department of Computer Science, University of Maryland, College Park, MD, 20742, United States of America; Adjunct Professor, UNITED STATES

## Abstract

The polymicrobial biofilm communities in Mushroom and Octopus Spring in Yellowstone National Park (YNP) are well characterized, yet little is known about the phage populations. Dominant species, *Synechococcus sp*. JA-2-3B'a(2–13), *Synechococcus* sp. JA-3-3Ab, *Chloroflexus sp*. *Y-400-fl*, and *Roseiflexus sp*. *RS-1*, contain multiple CRISPR-Cas arrays, suggesting complex interactions with phage predators. To analyze phage populations from Octopus Spring biofilms, we sequenced a viral enriched fraction. To assemble and analyze phage metagenomic data, we developed a custom module, VIRITAS, implemented within the MetAMOS framework. This module bins contigs into groups based on tetranucleotide frequencies and CRISPR spacer-protospacer matching and ORF calling. Using this pipeline we were able to assemble phage sequences into contigs and bin them into three clusters that corroborated with their potential host range. The virome contained 52,348 predicted ORFs; some were clearly phage-like; 9319 ORFs had a recognizable Pfam domain while the rest were hypothetical. Of the recognized domains with CRISPR spacer matches, was the phage endolysin used by lytic phage to disrupt cells. Analysis of the endolysins present in the thermophilic cyanophage contigs revealed a subset of characterized endolysins as well as a Glyco_hydro_108 (PF05838) domain not previously associated with sequenced cyanophages. A search for CRISPR spacer matches to all identified phage endolysins demonstrated that a majority of endolysin domains were targets. This strategy provides a general way to link host and phage as endolysins are known to be widely distributed in bacteriophage. Endolysins can also provide information about host cell wall composition and have the additional potential to be used as targets for novel therapeutics.

## Introduction

Polymicrobial biofilms are important in a variety of natural environments [1–3] as well as in clinical settings [4–6] and human microbiomes [7–9]. Biofilm forming communities are dynamic, with dense matrices and complex three-dimensional structures, often able to regenerate after perturbation [10,11]. Biofilms can display novel emergent properties not seen in individual cells or in monoculture, such as antibiotic resistance, production of toxins, resistance to chemical and/or physical disruption, phototaxis, and nitrogen fixation [12–16]. Biofilm members have co-evolved complex metabolic and physiological interactions between species, and spatial positioning within the matrix [17,18]. Such interactions are often tailored for specific environmental niches [19,20].

In an additional dimension of complexity, microbial biofilms also harbor phage populations that, in turn, have a significant impact on the entire community structure: exerting significant evolutionary selection, influencing metabolic capabilities and influencing overall growth and diversity [21–23]. Pioneering work by several groups [24–27] using high-throughput metagenomic DNA sequencing, metatranscriptomics and novel bioinformatics, have provided a tantalizing glimpse of extensive phage diversity from several natural environments.

Our knowledge of microbial communities in the alkaline siliceous hot springs of YNP is quite extensive at the biogeochemical, physiological, and more recently at the genomic/metagenomic level [28–31]. In contrast, information about the phage populations and their impact on microbial communities is much more limited [32,33]. Although this community is well suited to probe the dynamics of co-evolution of phage and microbial populations, availability of appropriate data has been lacking. Thus, our first objective was to build a database of phage DNA sequences (a virome) from photosynthetic microbial mats in YNP.

The important role of phage in the microbial mats of YNP is highlighted by the presence of the CRISPR-Cas (**C**lustered **R**egular **I**nterspaced **S**hort **P**alindromic **R**epeats, **C**RISPR **AS**sociated) adaptive immunity system in all three dominant phototrophs; *Synechococcus* sp. JA-2-3B'a(2–13) [CP000240], *Synechococcus* sp. JA-3-3Ab [CP000239], *Chloroflexus sp*. Y-400-fl [CP0Q1364] and *Roseiflexus* sp. RS-1 [CP000686]. While CRISPR-mediated adaptive immunity system is only one of many strategies used by cells to avoid phage attack [34] it is specific in linking host and phage relationships [32,35]. As new spacers are acquired into host CRISPR arrays at a certain rate and in a particular orientation, they are useful markers for analysis of host and co-existing phage populations [36,37]. Selection pressure is placed on the phage, to evade the host CRISPR defense system which relies on close nucleotide matching between acquired spacers and incoming phage sequence, and yet retain functionality [32,38].

A critical question to ask is if specific viral genes are preferentially targeted by the CRISPR-Cas system. Only a few studies have focused on CRISPR dynamics and viral targets in environmental settings [32,39–41]. Comparative analysis of CRISPR spacers in cyanobacteria using metagenomes derived from microbial mats in Octopus and Mushroom Spring and viromes derived from the source water of these hot springs suggested that spacers were being actively acquired by the host cyanobacteria, and could be used as a marker for host-phage interactions over short time intervals [28,32]. Initial environmental surveys also suggested that endolysins might play an important role in the YNP microbial mat communities [32].

We generated a virome from the top photosynthetic microbial mat layer of Octopus Spring using the 454 Titanium sequencing platform. Accurate assembly of phage sequence is challenging so we developed a custom strategy to utilize the assembled contigs and analyze host-viral co-evolution. A three-tier module, called VIRITAS, was developed to analyze phage metagenomic sequences. This module has been integrated into MetAMOS as a separate workflow (-W viritas) [42]. Using this pipeline, we assembled phage contigs, and binned related contigs by tetranucleotide analysis and CRISPR spacer matching. CRISPR spacer matching to cyanobacteria highlighted an endolysin domain: Glyco_hydro_108 (PF05838), prompting a characterization of the endolysin domains in OS-V-09 and sequenced cyanophages. We found that OS-V-09 contained only a subset of the annotated endolysins found in fully sequenced cyanophages. Led by these findings, we expanded our search and found that phage endolysins are a frequent CRISPR target. This allows for a general strategy to link unknown host and phage. This combination of widespread phage distribution and CRISPR spacer targeting suggest endolysins may be useful marker genes. Phage endolysins can be host species specific; they provide information about the host cell wall composition and can be harnessed as a useful tool for cell lysis, and have the potential to be used as candidates for novel therapeutics.

## Results and Discussion

### Generation of the OS-V-09 virome by 454 sequencing

**Fig 1:**
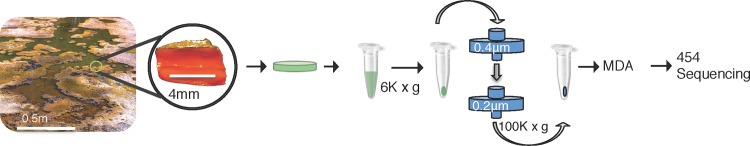
Generation of a Virome: OS-V-09. An 8mm mat core was excised from a microbial mat community in Octopus Spring, Yellowstone National Park. The top 1-3mm green layer was removed and re-suspended in Tris-EDTA buffer. Cells were pelleted and the supernatant passed sequentially through 0.4μm and 0.2μm filters. The filtered supernatant was pelleted via ultracentrifugation and subjected to MDA amplification with Phi29 polymerase. Amplified DNA was sequenced on the 454 Titanium platform.

A virome (hereafter referred to as OS-V-09: **O**ctopus**S**pring-**V**irome-20**09**) was generated from a phage-enriched fraction of a microbial mat core sample taken from a 60°C region in Octopus Spring, Yellowstone National Park. DNA was extracted and whole genome amplification (WGA; also termed Multiple Displacement Reaction (MDA) was required to ensure sufficient sample for sequencing (**Fig 1**). Prior to sequencing, putative phage primers designed from sequence generated by Schoenfeld et al. 2008 [33] herein named OS-V-03 and BP-V-03 (**S1 Table**), indicated phage DNA was present. The extent of bacterial DNA present in the virome was judged to be low based on the faint 16S rDNA signal that was found using general V1-V3 16S rDNA primers [43] in contrast to the robust 16S rDNA signal in whole mat DNA extractions (**S1 Fig**). A DNA sequence dataset of 180,141,543bp consisting of 501,240 reads was generated. Read distribution had a mean length of 359bp (longest read was 1385bp) and run statistics met or exceeded all quality control checks (**Table 1**).

**Table 1:** OS-V-09 454Ti Run Statistics.

Total Bases	180141543
Number of Reads	501240
Maximum Read Length	1385
Mean Read Length	359
Putative Bacterial Reads	116344
Putative Archaeal Reads	1864
Putative Phage Reads	383032

### Classification and identification of OS-V-09 reads

Reads generated from OS-V-09 were classified prior to assembly with VIRITAS via the Fragment Classification Program (FCP) [44]. Archaeal and Bacterial reads comprised 0.4% (1864 reads) and 23% (116344 reads) respectively, while the remaining reads 76.4% (383032 reads) had little to no homology to known bacterial or archaeal sequence (**Table 1**). Archaeal reads were predominantly Crenarcheota and Euryarchaeota, which are known to be ubiquitous in many environments, including hot springs [45,46]. The most numerous identifiable reads belonged to the bacterial phylum, Chloroflexi [18% (61,470 reads)] which are abundant in the mat and are more easily lysed than cyanobacteria [47–49]. Of the 501,240 reads from the virome, only 52 reads (0.01%) contained partial 16S rDNA sequences based on HMMER 3.0 predictions [50] Identified reads which spanned the 16S were aligned to known species (**S2 Fig**). This is comparable to the 24 reads (0.07%) found in OS-V-03 and BP-V-03 viromes which were treated with 10U benzonase endonuclease for 30mins [33]. The presence of such a low percentage of contaminating 16S rDNA sequences in the dataset provided further confirmation that OS-V-09 represented a dataset depleted for bacterial sequences and enriched for phage sequences.

### Assembly of phage reads with the VIRITAS pipeline

**Fig 2:**
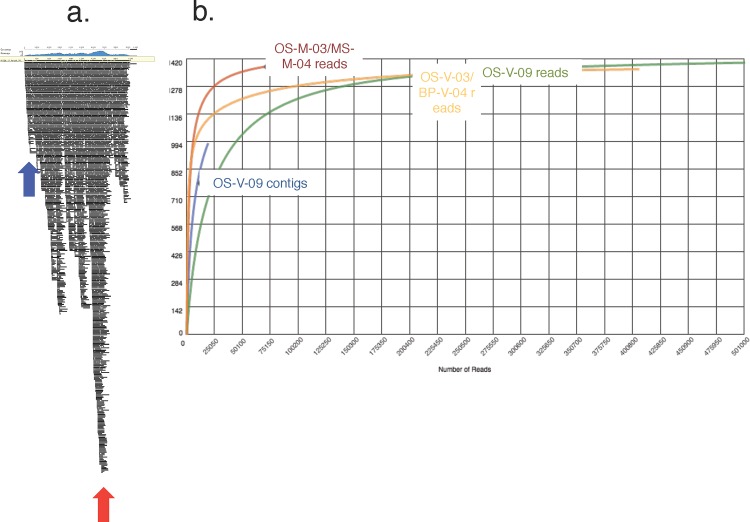
Assembly of a Virome. A) A typical assembled viral contig (length 7002bp) showing a region of low coverage (blue arrow) and a region of high coverage (red arrow). B) A rarefaction curve generated in MG-Rast [55] showing metagenomic reads from OS-M-03 and MS-M-04 (red line), viral metagenome reads from OS-V-03 and BP-V-04 (yellow line), assembled contigs from OS-V-09 (blue line) and metagenomic reads for OS-V-09 (green line).

In an attempt to mitigate the challenges of *de novo* viral assembly [51,52] and MDA bias typical of Phi29 polymerase activity [53] while producing high quality contigs, we employed the SPADes assembler within the VIRITAS pipeline to assemble reads [54]. We were able to recruit 99.8% of the reads (500,128 of a total of 501,240 reads) in the final assembly (**Table 2**). A total of 19,837 contigs were assembled, with an N50 value of 605bp (**Table 2**). As expected, we observed an uneven read coverage of assembled contigs such that some regions were over-represented (345x coverage) or under-represented (25x coverage) (**Fig 2A**) which is typical of the activity of Phi29 polymerase [53].

**Table 2:** SPADes Assembly Statistics.

Reads Assembled	500128
# contigs	19837
# contigs > 200bp	19091
Largest contig	16155
GC (%)	47.2
N50	605
N75	452
L50	4798
L75	10671

Assembled contigs were run through MG-RAST [55] to generate a rarefaction curve. For comparison, metagenomic reads from Octopus and Mushroom Spring (OS-M-03 and MS-M-04), phage metagenome reads from OS-V-03 and BP-V-04, and the unassembled viral reads for OS-V-09 were also plotted (**Fig 2B**). Although OS-V-09 *reads* start to reach saturation, we clearly observe that the assembled OS-V-09 *contigs* are still in the exponential phase of the curve. This reflects what we would expect with Phi29 polymerase amplified sequences; the high coverage bias produces an artifact which suggests that saturation has been reached, as individual reads may oversample the same sequence.

### Binning of phage contigs by tetranucleotide analysis, and CRISPR spacer matching followed by visualization using Emergent Self Organizing Maps (ESOMs)

To bin contigs, which did not assemble into a consensus sequence, yet may have come from related phage, we used tetranucleotide frequency analysis (TNF) which has been successful in binning sequences from isolated genomes, metagenomes, as well as prophages [22,39,56]. In TNF analysis many data points can be collected and are less likely to be affected by overall genome GC content, or nucleotide biases [57]. Only contigs greater than 1Kb in length, were clustered via TNF scripts [57], as the accuracy with which sequences are correctly assigned is correlated with contig length [58]. The frequency of the 256 tetramers (136 non-redundant) was calculated for viral reads as well as several well-characterized microbial mat members: *Synechococcus* sp. JA-2-3B'a(2–13), *Synechococcus* sp. JA-3-3Ab, *Meiothermus silvanus*, *Chloroflexus sp*. Y-400-fl and *Roseiflexus* sp. RS-1 and visualized as a heat map (with red indicating low frequency and yellow indicating high frequency) (**S3 Fig).** Viral contigs fell into distinct clusters; with some viral reads containing a fingerprint very similar to known bacterial genomes, while other contigs had a very unique pattern not associated with a known genome.

**Fig 3:**
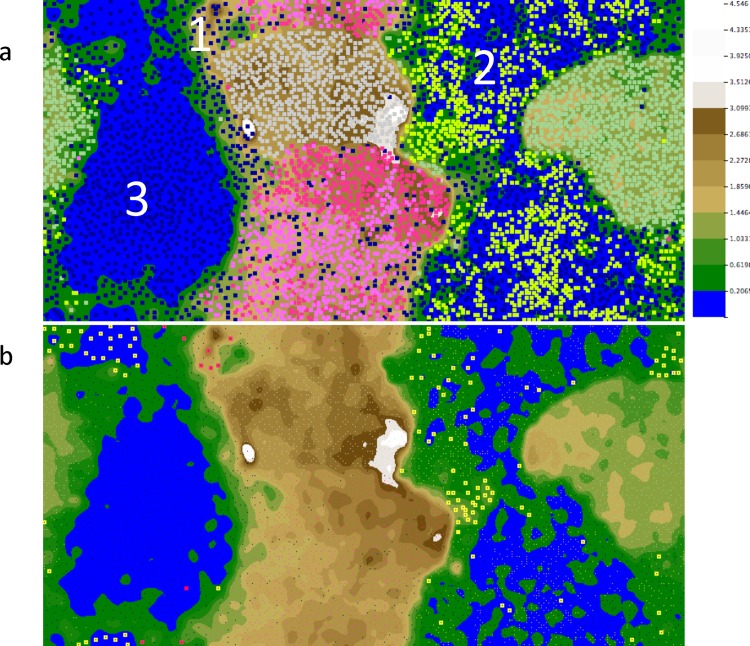
ESOM of Assembled Viral Contigs. A) The tetranucleotide signature for viral contigs greater than 1Kb (navy), as well as 5K fragments from five genomes from fully sequenced mat species *Synechococcus* sp. JA-2-3B'a(2–13) (light pink), *Synechococcus* sp. JA-3-3Ab (salmon pink), *Meiothermus silvanus* (light grey), *Chloroflexus sp*. Y-400-fl (mint green) and *Roseiflexus* sp. RS-1 (yellow), was calculated via scripts from Dick et al [59]. Viral contigs clustered into three major groups (Cluster 1–3). B) Viral contigs with at least one CRISPR spacer hit re-coloured to reflect their host as shown in part a. Legend represents tetranucleotide frequency distances from valleys (blue) to peaks (white).

To more clearly visualize bins, calculated TNF was input into the ESOM-Mapping tool [59] (**Fig 3A**). The five genomes (*Synechococcus* sp. JA-2-3B'a(2–13), *Synechococcus* sp. JA-3-3Ab, *Meiothermus silvanus*, *Chloroflexus sp*. Y-400-fl, and *Roseiflexus* sp. RS-1 could be clearly separated into distinct clusters, as expected. In the ESOM map, we could also clearly visualize the viral contigs. The 2052 viral contigs (above 1Kb) fell into three main clusters: Cluster 1 included 171 viral contigs that were associated with the two *Synechococcus* genomes, Cluster 2 included 1175 contigs intermixed with the *Roseiflexus* RS-1 genome, and Cluster 3 included 706 viral contigs that were not closely associated with any host genome. In contrast, only a few viral contigs were associated with the *Meiothermus silvanus* or *Chloroflexus sp*. Y-400-fl genome clusters.

Next, to determine putative host-phage pairs, CRISPR spacer-protospacer matching information from dominant species present in the mat was overlaid on the ESOM maps. First, all CRISPR spacers from CRISPRdb in addition to spacers manually extracted via CRISPRfinder from relevant environmental datasets (**S2 Table**) were blasted against assembled viral contigs. We were able to identify the host for the three distinct clusters, labeled Cluster 1–3 (**Fig 3A, S3 Table**). Cluster 1 had 13 contigs which contained matches to Cyanobacterial CRISPR spacers. Cluster 2 had 116 contigs with CRISPR spacer hits to *Roseiflexus* spacers. Cluster 3 had only one CRISPR spacer match to a Cyanobacterial spacer. To visualize this subset of contigs with CRISPR spacer matches more clearly, viral contigs with at least one spacer hit are shown in **Fig 3B,** coloured to match their putative host. By using tetranucleotide binning in parallel with CRISPR spacer matching and ESOM visualization, we consolidated the dataset, grouping sequences which were not assembled, but which retained similar signatures, and identified the predicted hosts.

We identified a total of 1546 spacers, which included the spacers from *Synechococcus sp*. *JA-2-3B'a(2–13)* {125 spacers} and *Synechococcus sp*. *JA-3-3Ab* {96 spacers} genomes and a further 1325 spacers that were manually identified from metagenome and virome reads (**S**2 **Table**). If we assume that on average, an individual *Synechococcus* cyanobacterium has ~100 unique spacers, then this spacer database is representative of a sample size of only 15 individuals. This emphasizes that without further expansion of the cyanobacterial CRISPR spacer database, conclusions regarding spacer acquisition dynamics will be limited.

### Identification of predicted ORFs and those containing CRISPR spacer hits in assembled contigs

**Fig 4:**
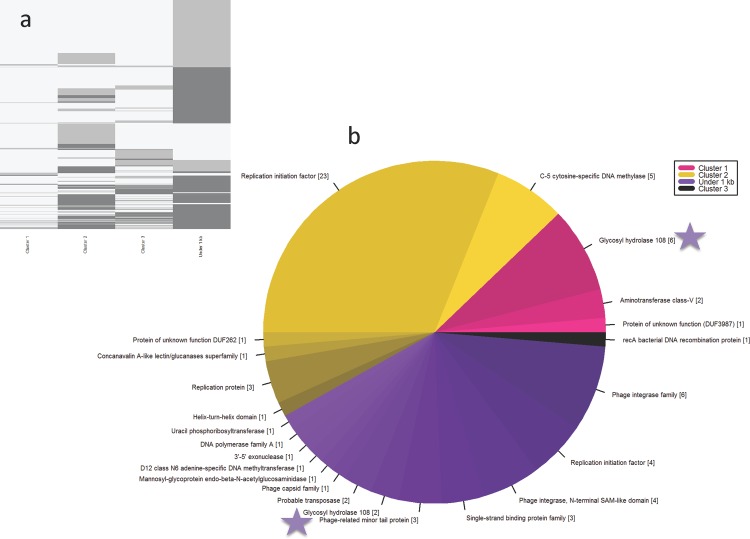
Breakdown of ORFs Containing CRISPR Spacer Matches by Bin. A) Pfam distribution across Clusters 1–3 and contigs under 1Kb visualized as a heat map. Colour corresponds to count, with black = 0, medium grey = 1, and light grey = 2 or more. B) ORFs with known predictions containing CRISPR spacer matches from contigs over 1Kb (Cluster 1, 2 & 3) as well as under 1Kb (shown in purple). Glyco_hydro_108 domains are marked with a purple star.

ORFs were predicted in the assembled contigs via ORFfinder [60] with a minimum size of 300bp, and run through InterProScan [61] to detect identifiable domains (**S4 Table**). A total of 52,348 ORFs were identified (getorf -minsize 300), of which 9319 (i.e. 17.8%) contained domains identifiable by Pfam. As expected, a majority of predicted ORFs did not have any recognizable domain, which is a common feature of viral datasets. However, some ORFs were predicted to be of phage origin based on their annotation; including phage portal proteins, terminases, VirE and integrases, as well as host genes frequently observed to be carried by phage: methyltransferases, the most common gene observed in environmental phage enrichments [62] and PAPS_reductase (thioredoxin), an essential enzyme in prokaryotic sulfur assimilation pathways known to be carried in sequenced cyanophages [63]. To visualize ORF distributions across clusters, we generated a heat map based on all identified Pfam annotations to look for broad-scale similarities and differences (**Fig 4A)**. Pfams with no hits are shown in light grey, while Pfams with 1 representative are shown in medium grey and those with 2 or more are shown in dark grey. We observe that individual bins share common Pfams (such as phage integrases and methyltransferases) while other domains are unique per cluster.

Identifiable ORFs containing CRISPR spacer matches were broken down by cluster (**Fig 4B).** Cluster 1 contained 188 domains identified via Pfam, nine of which contained CRISPR spacer matches to 3 unique domains. Cluster 2 contained 2314 domains characterized by 34 *Roseiflexus* CRISPR spacer hits to 6 unique domains. Cluster 3 contained 971 domains, with 1 CRISPR spacer hit. Contigs under 1Kb contained 5826 domains with 30 CRISPR spacer hits from *Roseiflexus*, *Chloroflexus* and *Synechococcus* to 13 unique domains.

**Fig 5:**
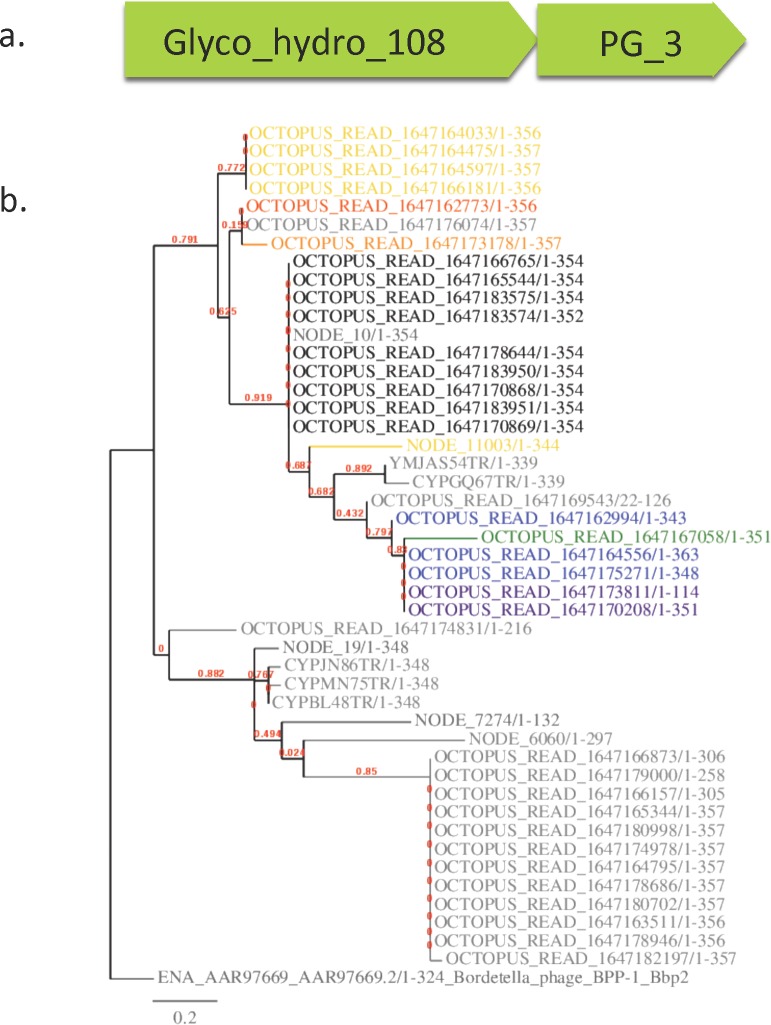
Survey of Glyco_hydro_108 binding domains. A) Glyco_hydro_108 and PG_3 domain organization b) Glyco_hydro_108 nucleotide domains (PF05838) from OS-V-09 (indicated by the prefix “**NODE**”), MS-M-04/OS-M-03 (indicated by prefixes YMJ and CYP), and OS-V-03 (indicated by the prefix “OCTOPUS_READ”) in addition to outgroup Bordetella_phage_BPP-1 were identified via HMMsearch, aligned with MUSCLE, and the gene tree visualized using the MABL server (phylogeny.fr). Overlaid on the protein tree are significant nucleotide hits (greater than 70% ID over 85% length) to cyanobacterial CRISPR spacer CRISPR_II_YMBCR81TF-SP-2 (previously shown to target a Glyco_hydro_108 domain [Heidelberg, 2009]) as determined by BLASTn. Hits are visualized via text colour corresponding to % hit identity. (95% purple, 92% blue, 86% green, 80% yellow, 75% orange, 73% red, 70% black, grey = no hit). In (A) closely related sequences are denoted from the same dataset with a similar hit identity. In (B) sequences with recognizable Glyco_hydro_108 domains have different identity spacer hits.

Most CRISPR spacers mapped to hypotheticals or proteins of unknown function. A notable exception was the endolysin Glyco_hydro_108 (PF05838) and the closely associated PG_3 binding domain (**Fig 5A**). We focused on this domain for two reasons. First, it served as a test case to determine genomic diversity in the phage population, since each read represents the genome within an individual viral particle. Second, it allowed us to explore the potential for using it as a phage marker gene and identifying strategies for the identification of additional useful phage marker genes, as only a limited number of these have been established [64].

### Identification of Glyco_hydro_108 domains

We identified 47 full length open reading frames that included the Glyco_hydro_108 (PF05838) and PG_3 (PF09374) domains from reads in several relevant datasets. These included previous YNP microbial mat metagenomes from Octopus and Mushroom Springs, a 93°C virome from Octopus and Bear Paw Springs, and the 60°C virome we generated from Octopus Spring (**S1 Table**). Sequences were aligned via Muscle in Jalview [65] (**S4 Fig**) and the phylogenetic relationship visualized using the MABL server [66]. Significant CRISPR spacer hits to cyanobacteria spacer YMBCR81TF_sp_2 were represented on the tree as coloured text to represent varying degrees of nucleotide identity (**Fig 5B**). This allowed us to make the following observations. First, within these datasets, we observe a range of spacer hit identities between 70–95%. We also observed high identity CRISPR spacer hits in data collected in 2003 as well as in 2009. The same sequences are present over a 6-year span that may indicate rapid turn-over rates, or reflect phage sequence persistence. Second, high percentage identity CRISPR spacer hits are found in several tree branches, and are not strongly correlated with either protein relatedness, sample location, or year the sample was taken. Third, we found recognizable Glyco_hydro_108 domain variants present in the dataset, yet not all contain a cyanobacterial CRISPR spacer hit. This could be because we have not reached saturation in cyanobacterial CRISPR spacer sequence databases or that these are endolysins present in phages that target other host species.

### Endolysin Distribution in OS-V-09 Assembled Contigs

The additional four contigs containing Glyco_hydro_108 domains did not have CRISPR spacer hits to any known species, and could not be assigned a putative host (**S4 Table**). The presence of these untargeted endolysins might indicate that we have do not have adequate spacer coverage in hosts. Phage fecundity highly depends on successful host lysis, thus endolysins may be preferred targets of the CRISPR system as they are also under strong evolutionary pressure [67], making them an efficient target.

### Phage endolysin domains present in OS-V-09 as compared to annotated cyanophages

**Fig 6:**
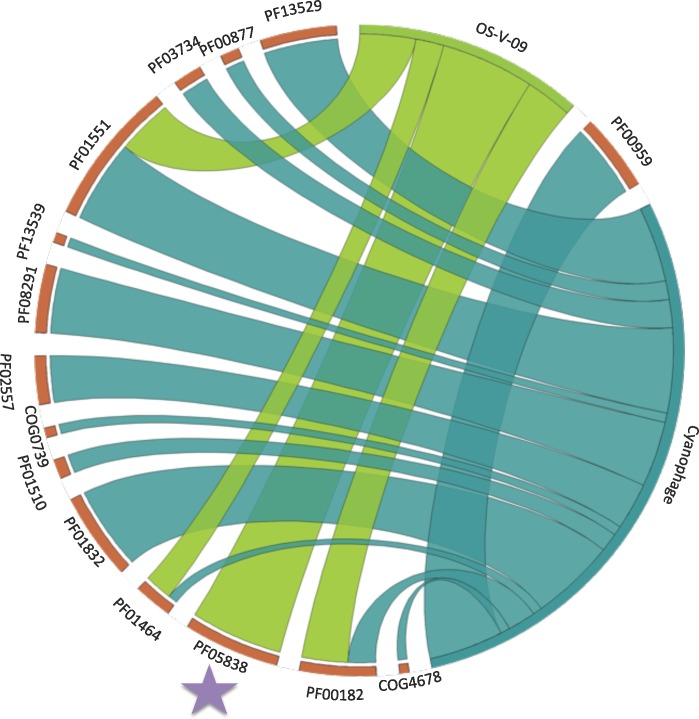
Distribution of Endolysin Catalytic Domains in Sequenced Cyanophages and OS-V-09. CIRCOS plot depicting the distribution of Endolysin Catalytic Domains (shown in red) found in OS-V-09 (shown in green) and in annotated cyanophage genomes from IMG (shown in teal). A subset of domains (PF00182, PF05838, PF01464 and PF01551) were found in thermophilic phage as compared to cyanophages. In addition, the Glyco_hydro_108 (PF05838) indicated with a purple star was only found in OS-V-09.

To determine if endolysins can potentially be used as a phage marker gene, similar to the use of 16S rDNA to identify bacterial phyla, we identified phage endolysin domains across all sequenced cyanophage retrieved from JGI DOE IMG (img.jgi.doe.gov, last update June 2015, 3899 phages, 68 cyanophages). Endolysins are typically composed of two domains; a catalytic domain followed by a binding domain. These domains are modular, and can be found in multiple combinations [68]. For OS-V-09 contigs, OS-M-03/OS-M-04 reads, and metagenome reads, open reading frames greater than 300bp were identified by getorf, part of the EMBOSS software package [69], and searched with Markov Models via HMMsearch (**S5 Table**). There are 14 endolysin domains in cyanophage (**S6 Table**). We observed that only a subset of four catalytic endolysin domains (PF00182, PF05838, PF01464 and PF01551) overlapped between OS-V-09 and annotated cyanophages (**Fig 6**). Of note, the Glyco_hydro_108 (PF05838) domain was not found in previously sequenced cyanophages. This might suggest that endolysins are predictive of a particular phage-host lifestyle or environment, and could be useful as a diagnostic for host-phage relationships.

### CRISPR targeting of phage endolysins is not exclusive to cyanobacteria

To determine if CRISPR spacer targeting of phage endolysins is a general phage strategy, or specific to cyanobacteria, a BLASTn was run with all known spacers in CRISPRdb against all annotated phage endolysin domains (as identified Olivieria et al 2013 [68]) in IMG. Significant hits (90%ID, evalue = e-5) were mapped to HMM logos [70] (**Table 3**). We observed that most phage endolysin domains contained CRISPR spacer domains, and that in some cases they are heavily targeted while a few domains have none (although this could be due to underrepresentation of CRISPR spacers). For comparison, we also analyzed a few other phage genes. VirE and a phage portal gene also contained some CRISPR spacer hits, although NinC a non-structural gene had no CRISPR spacer hits. As CRISPR spacer databases and phage databases get bigger, it will be possible to extend such studies to examine if there are preferred targets of the CRISPR spacer immunity system.

**Table 3:** Endolysin CRISPR hit distribution.

IMG annotated domains	Number of annotated domains with hits	Annotation	Percentage of domains with a hit
292	6	Phage_lysozyme (LYSO)	2.054794521
46	1	Muramidase (MURA)	2.173913043
41	1	Glyco_hydro_19 (GH19)	2.43902439
53	4	Glyco_hydro_25 (GH25)	7.547169811
36	1	Glyco_hydro_108 (GH108)	2.777777778
168	10	SLT	5.952380952
20	1	Transglycosylase (TRANG)	5
92	18	Glucosaminidase (GLUCO)	19.56521739
10	1	Amidase02_C (AMI02-C)	10
23	11	Amidase_5 (AMI-5)	47.82608696
81	7	Amidase_3 (AMI-3)	8.641975309
197	0	Amidase_2 (AMI-2)	0
5	0	NlpD (NLPD)	0
9	4	VanY (VANY)	44.44444444
2	0	Peptidase_U40 (PET-U40)	0
36	1	Peptidase_M15_3 (PET-15-3)	2.777777778
103	5	Peptidase_M15_4 (PET-15-4)	4.854368932
91	2	Peptidase_M23 (PET-M23)	2.197802198
3	2	YkuD (YKUD)	66.66666667
131	4	NLPC_P60 (NLPC-P60)	3.053435115
17	7	Peptidase_C39_2 (PET-C39-2)	41.17647059
175	21	CHAP	12
2	0	DUF3597 (DUF)	0
33	0	PG_binding_3 (PG-3)	0
82	4	LysM (LYSM)	4.87804878
9	1	SH3_3 (SH3-3)	11.11111111
93	5	SH3_5 (SH3-5)	5.376344086
87	1	PG_binding_1 (PG-1)	1.149425287
0	0	ChW (CHW)	0
4	1	Cpl-7 (CPL7)	25
5	2	LGFP	40
0	0	SH3-related (SH3-r)	0
4	0	FOG	0
7	2	SPOR	28.57142857
0	0	SLAP	0
266	22	portal	8.270676692
53	15	virE	28.30188679
30	0	NinC	0

## Conclusions

### Co-evolution of Host and Phages in YNP communities

The alkaline siliceous hot springs of YNP have been extensively analyzed at the biogeochemical, physiological, and at the genomic/metagenomic level and provide a good model system in which to examine host-phage co-evolution dynamics [28–31]. The comparative genomic analysis of two *Synechococcus* species isolated from different temperature regions of Octopus Springs in YNP revealed that both contained CRISPR-Cas systems [32]. Two distinct CRISPR types, distinguished by their repeat sequences, were common to both genomes, although the spacers were unique in each genome. The genome of *Synechococcus* OS-A contained an additional third CRISPR type that appeared to be shared with other microorganisms that inhabit the mat and may have undergone horizontal gene transfer [32].

Comparative analysis of CRISPR spacers in cyanobacteria using metagenomes derived from microbial mats and viromes derived from the source water of these hot springs suggested that spacers were being actively acquired by the host cyanobacteria, and could be used as a marker for host-phage interactions over short time intervals [28,32]. In particular, a few host spacers matched regions of a putative viral lysozyme/endolysin suggesting that host spacer matches to viral sequences could be a powerful way to characterize putative viral-host relationships as well as gain insight into the strategies used by host to avoid phage attack and conversely to explore how phages evolve to evade host defenses. The microbial mat community is well suited to probe the dynamics of co-evolution of phage and microbial populations, but availability of appropriate data has been lacking [32,33]. Thus, our first objective was to build a database of phage DNA sequences (a virome) from the photosynthetic microbial mats in YNP.

We observed that CRISPR spacers matches were highly conserved even across a span of 6 years. Spacers curated from metagenomic sequence collected in 2003 contained hits to a 2009 virome with high conservation (**Fig 5**). Second, high percentage identity CRISPR spacer hits are found in several tree branches, and are not strongly correlated with either protein relatedness, sample location, or year the sample was taken. This could be explained by either very high or moderate CRISPR turn-over rates. To quantify these rates in a natural environment, our results suggest a time series with both monthly and yearly times scales would be most informative.

### Technical challenges in phage genome assembly

*De novo* assembly of metagenomic sequences, in particular, phage-derived sequences, is a challenging computational task. In spite of recent technological advances, such as preassembly read-filtering by digital normalization and partitioning [71], and use of a variety of sequencing platforms to minimize the shortcomings of any one technique [72], reconstructing an entire genome, from a metagenome or virome sequence database remains an open problem [73]. In this environmental biofilm many genomes of highly similar strains are present and evidence suggests recombination is occurring at a high rate [28,74]. Such high strain level diversity can cause assemblers to fail or result in hybrid assemblies combining variations found in several similar species or strains. In contrast, highly conservative assemblers will break the assembly at regions of variation, which results in highly fragmented, non-cohesive assemblies. A further complication results from amplification artifacts introduced by Phi29 polymerase during MDA, yielding uneven coverage, breaking multiple assembly heuristics for resolving repeat structure, or resulting in chimeric reads [51,52].

By using SPADes, an assembler specifically tuned to address MDA artifacts, we were able to create robust viral assemblies. Binning of the contigs via tetranucleotide analysis and visualization by ESOM enabled us to group sequences and also allowed us to characterize the phage types present within the dataset. We were encouraged by the fact that clustering of viral contigs to host was robust and was corroborated by CRISPR spacer matching. This strategy was independent of the system used, and thus represents a general pipeline for viral sequence analysis. Furthermore, CRISPR spacer matches provided insight not only as to the host, but also into which ORFs were targeted. As our analysis was built upon datasets generated over the course of several years, we were able to observe CRISPR spacer turn-over. Although we only had a few “snap-shots” of sequence, a deeper targeted time-course dataset would allow us to differentiate between rapid or moderate turn-over in a natural environment. Further expansion of the cyanobacterial CRISPR spacer database will be required to get more insight into spacer acquisition dynamics.

### Identification of phage proteins

Phage proteins are notoriously difficult to identify, often with no known homologues in sequence databases; the so-called viral “dark matter” [75–77]. Consequently, most phage genes are still annotated as “hypothetical” or of “unknown function” [57,78]. In contrast to bacteria, no universally conserved gene (such as the 16S rDNA) exists in phage, hindering attempts to identify phage genomes or survey abundance in natural environments [79]. In cyanophage, structural markers such as capsid, portal, or tail sheath proteins have been used to determine viral abundance across time and sampling locations, while ribonucleotide reductases have been recently posited as a phage marker candidate with a broad host range [80]. Tracking dynamics of these genes allows for inference of the viral impact on host and the frequency at which host cells are infected [81,82]. However, using a single marker gene approach, has several drawbacks: sequences that are divergent, or have undergone inter or intra-genic recombination may not be identified, even with degenerate primers; PCR amplification may introduce biases, so that the amplified genes are not representative of the natural population distribution; rare phage sequences may not be amplified at all. One means of mediating these shortcomings is the use of a “panel” of phage gene markers [83].

### Endolysins as useful phage marker genes

Endolysins make an attractive candidate to add to the panel of phage marker genes. Endolysins are highly specialized, exquisitely timed hydrolytic components involved in successful release of phage particles from infected cells, and have been recently characterized for all double-stranded sequenced bacteriophage [67,68]. Endolysin classification is dependent on their mode of action, with four types discovered to date: lysozymes and transglycosylases cleave glycosidic bonds between amino sugars in the cell wall, while amidases and endopeptidases cleave crosslinking oligopeptide bonds [68].

Endolysins contain regions of high conservation, as well as variable regions [68], not unlike the golden standard of 16S rDNA used for bacteria. Sequences containing regions of conservation allow for robust assemblies, even of highly diverged variants, while the variable regions allow for fine scale resolution. In addition to yielding information about the phage, endolysins also simultaneously reveal information about their host specifically about the cell wall composition.

We show that endolysins are frequently targeted by spacers. Experiments under laboratory conditions have shown that CRISPR spacers can be enriched for specific gene targets, in particular, an endolysin domain was found to be over-represented as a spacer target [37]. In this study we show that in a natural community, phage endolysins were targeted by the host and this may represent a general strategy in host-phage interactions (**Table 3**) although further analysis will be required to establish that this is a common mechanism.

### Practical applications of Endolysins

The role that endolysins play in creating and maintaining a biofilm is not straightforward. Lysins can be key factors in helping to prime biofilm formation, by producing an initial extracellular DNA scaffolding, which is later predominantly replaced by exopolysaccharides [84]. However, timing is crucial, as lysins can also destroy a more mature biofilm [85]. Such a delicate balance in timing adds a further layer of complication to the host-phage relationship. Many species are pathogenic only in a biofilm state, and endolysins represent a potential novel source of antimicrobials, effective against infections which may be resistant to antibiotics [86]. Some endolysins display broad-host ranges, while others have “near-species specificity” of domains [87]. Species specificity can also be engineered, with inducible lysins specifically targeted to particular species for optimal breakage, or transformation, such as cyanobacterial targeted via a green light inducible T4 phage holin/endolysin [49]. Endolysins can also be targeted to disease causing members of a community, while leaving the remaining consortia intact. This strategy was effective with targeting of *Clostridium* with a bacteriophage endolysin delivered via probiotic species [88,89]. Lastly, while endolysins can diffuse freely across the cell membrane of gram negative species, “artilysins” i.e. endolysins that have been have also been engineered to target the outer membrane of gram negative species, have also shown great promise as novel antibiotics [90].

## Materials and Methods

### Generation of Viral DNA Sequence (Virome; OS-V-09)

The uppermost 1-2mm green layer was excised from a 2009 microbial mat core sample (8mm diameter) from Octopus Spring (stored at -80°C until use) and re-suspended in 50 mL 10mM Tris, 1mM EDTA by vigorous vortexing. Cells were pelleted at 6000 x g for 10 minutes in a Sorvall GS5C. The supernatant was passed sequentially through 0.45μm and 0.2μm filters (Nalgene, Thermo Scientific) to remove remaining intact cells and any cellular debris. One mL aliquots were centrifuged at 50,000K (Beckman TL-100 Ultra Centrifuge, TLA 100.3) for one hour to concentrate viral particles. Viral DNA was amplified from these enrichments via a Phi29 polymerase (GenomiPhi, GE) in two independent technical replicates (**Fig 1**). Amplified viral DNA was subjected to a panel of *Syn* OS-BSand *Syn* OS-A specific primers and universal bacterial 16S primers for the V1-V3 variable regions [91]. Putative viral primers designed from sequence generated by Schoenfeld et al. 2008 [33] herein named OS-V-03 and BP-V-03 (**S1 Table**), indicated an enrichment of viral sequences (**S1 Fig**). To reduce random biases, two technical replicate MDA reactions were pooled and sent for sequencing with 454 Titanium technology at the Genome Sequencing and Analysis Core Resource (http://genome.duke.edu/cores/sequencing/ at Duke University) resulting in a DNA sequence database of 180,141,543bp, consisting of 501,370 reads, with a read distribution median length of 425bp (the longest read was 1385bp, and the shortest was 40bp) and run statistics met or exceeded all quality control checks (**Table 1**). This dataset was named OS-V-09.

### Identification of CRISPR arrays and spacers in sequenced genomes and in OS-V-09

CRISPR repeat sequences were identified in fully sequenced genomes of *Synechococcus* sp. JA-2-3B'a(2–13) (NC_007776), *Synechococcus sp*. JA-3-3Ab (NC_007775), *Roseiflexus sp*. RS-1 (NC_009523), *Roseiflexus castenholzii* DSM 13941 *(*NC_009767), *Chloroflexus sp*. Y-400-fl (NC_012032), *Chloroflexus aggregans* DSM 9485 (NC_011831), and *Chloroflexus aurantiacus* J-10-fl (NC_010175) via CRISPRdb [92] and compared to OS-V-09 with Standalone BLASTN [93]. As the virome included reads with homology to bacterial sequences, we scanned the dataset for the possible presence of CRISPR spacers and repeats. Reads containing at least three repeat motifs were pipelined through CRISPRfinder [94] to extract potential CRISPR spacers using the default parameters. Results were manually inspected to remove any spurious spacer calls, such as repeat-rich sequences, that are not associated with CRISPR loci. A total of 38 were identified: 2 spacers on reads containing cyanobacteria-like repeats, 33 spacers on reads also containing *Roseiflexus* sp. repeat sequences, and 3 spacers from reads also containing *Chloroflexus* sp. repeat sequences (**S2 Table**).

### Identification of CRISPR arrays present in Metagenome Reads and Extraction of Novel CRISPR spacers

To identify CRISPR arrays in previously generated thermophilic microbial mat datasets, MS-M-04, OS-M-03, OS-V-03, BP-V-03, LIBGSS_012136, and LIBGSS_012135, were pipelined through CRISPRfinder and identified repeats were subjected to a BLASTN against the nr database to determine the species from which they originated [95]. Identified spacers were manually inspected to remove any spurious spacer calls, such as repeat-rich sequences, that are not associated with CRISPR loci. A total of 1546 spacers were identified with *Synechococcus* sp.-like repeats, and a total of 2828 spacers with *Roseiflexus* sp.-like repeats, and 1455 spacers with *Chloroflexus* sp.-like repeats from these datasets. (**S**2 **Table**).

### CRISPRs collected from CRISPRdb

CRISPR spacers were downloaded from CRISPRdb (Last update 2014-08-05)

### Assembly of viral reads with SPADes

OS-V-09 reads were fragmented *in silico* into 100bp fragments (from both the left and right) in preparation for input into SPADes3.7.1. Any “reads” smaller than 100bp were discarded. (—only-assembler—s1 OS-V-09 –sanger OS-M-04).

### Mate-pair read recruitment

To mine all available information from previously published sequences, we recruited mate-pair reads from similar environments: MS-M-04, OS-M-03, OS-V-03, BP-V-03 (**Table 1**) in an attempt to generate additional scaffolds. The majority of the recruited mates validated the assembled contigs, but did not extend contig length or assemble into new contigs.

### Rarefaction

Rarefaction curves were generated in MG-RAST [55] via blastn against GenBank using a maximum e-value of 1e-5, a minimum identity of 60%, and a minimum alignment length of 15aa.

### Phage annotation pipeline

Getorf part of the EMBOSS software package [69] was used to extract open reading frames over 300bp in length (getorf–minsize 300). Predicted open reading frames were pipelined through InterproScan [61] to identify recogniseable domains.

### Tetranucleotide Analysis and Emergent Self Assembling Map generation

Tetranucleotide frequency was calculated using scripts from Dick et al (https://github.com/tetramerFreqs/Binning) [57] from assembled contigs larger than.1Kb in length (number). Contigs less than 1Kb often result in “noisy” signatures and were excluded from further analysis. The gplots heatmap.2 R function was then utilized to generate the heat map based on hierarchical clustering of tetranucleotide frequency of the assembled contigs. Hclust function was used to order the tree diagram through the distance between the rows. (**S3 Fig**). Emergent Self Assembling Maps (ESOM) were created to better visualize the clustering within the viral dataset. The ESOM was anchored by including several known genomes of organisms found in the microbial mat community, namely, *Synechococcus* sp. JA-2-3B'a(2–13) (NC_007776), *Synechococcus sp*. JA-3-3Ab (NC_007775), *Roseiflexus sp*. RS-1 (NC_009523), *Chloroflexus sp*. Y-400-fl (NC_012032), and *Meiothermus silvanus* (NC_014212).

### Mapping of CRISPR Spacers onto Assembled Contigs

CRISPR spacers were BLASTed against assembled contigs (**S3 Table**). Matches were considered significant greater than e-5 for 90% [personal communication, David Paez]

### Analysis of Glyco_Hydro_108 domains in OS-V-09

Glyco_Hydro_108 (PF05838) domains were identified in seven assembled viral contigs with HMMSEARCH [50]. Contigs were aligned with Muscle [96].

### Distribution of Glyco_hydro_108 domains in relevant datasets

Full length open reading frames including the Glyco_hydro_108 (PF05838) and PG_3 (PF09374) domains from relevant datasets (**S1 Table**) were aligned via Muscle in Jalview [65]. Trees were visualized with the MABL server (phylogeny.fr). Significant CRISPR spacer hits were overlaid as coloured dots or bars to indicate CRISPR spacer hits analyzed from Heidelberg et al 2009 [32] with varying degrees of nucleotide identity (**Fig 4**).

### OS-V-09 contains a subset of known endolysin catalytic domains in sequenced cyanophages

Endolysin domains of interest for sequenced genomes (as characterized by Oliveira, et al [68]) were retrieved from pre-computed functional annotation with HMMER 3.0 in IMG. For OS-V-09, all open reading frames were extracted with getORF (-minsize 300) via command line. Hmmsearch (defaults) was used to search sequences with raw HMM models (pfam.xfam.org). Counts are shown in **S6 Table**. Plots were generated in Circos (http://mkweb.bcgsc.ca/tableviewer/).

### CRISPRdb spacer hits to annotated Glyco_hydro_108/PG_3 domains

CRISPRdb spacer hits to annotated Glyco_hydro_108 and PG_binding domains were retrieved from IMG via the find function option [97] (**Fig 6**) and blastn (ID = 90%, evalue = e-6) against all spacers from CRISPRdb.

## Supporting Information

S1 Fig Presence of 16S sequence in the viral MDA preparation. Viral reads present in OS-V-03 and BP-V-03 were used to generate viral specific primers (wells 40–58). General bacterial 16S RNA primers V1for and V3rev [91] were used to amplify a 460bp fragment. An intense16S band is observed in Mat DNA, while the amount of 16S present in the viral MDA prep is very faint.
(TIFF)

S2 Fig 16S phylogeny of bacterial reads in OS-V-09. Phylogeny of twenty-six identified 16S viral reads with known organisms. Cyanobacteria are marked in green, while Chloroflexii in orange.
(TIFF)

S3 Fig Tetranucleotide Analysis as visualized via a heat map. Contigs greater than 1Kb were pipelined through custom scripts by Dick et al [57] to calculate tetranucleotide frequency.
(TIFF)

S4 Fig Nucleotide alignment input for Fig 5B. (TIFF)

S1 Table Datasets Generated or Used in this Study. (CSV)

S2 Table Sources of Identified CRISPR spacers. (CSV)

S3 Table CRISPR spacer hits to contigs. (CSV)

S4 Table Predicted ORFs with PFAM annotations. (TSV)

S5 Table Contigs containing predicted Glyco_hydro_108 domains. (CSV)

S6 Table Endolysin domains in Annotated Cyanobacteria, Phage and Relevant Datasets. (CSV)

## Abbreviations

CRISPR: **C**lustered **R**egularly-**I**nterspaced **S**hort **P**alindromic **R**epeats
MDA: Multiple Displacement Amplification
NGS: Next Generation Sequencing

## References

[1] Hua Z-S, Han Y-J, Chen L-X, Liu J, Hu M, Li S-J, et al Ecological roles of dominant and rare prokaryotes in acid mine drainage revealed by metagenomics and metatranscriptomics. ISME J. 2014;9:6:1280–94 10.1038/ismej.2014.212 25361395 PMC4438317

[2] Jones DS, Albrecht HL, Dawson KS, Schaperdoth I, Freeman KH, Pi Y, et al Community genomic analysis of an extremely acidophilic sulfur-oxidizing biofilm. ISME J. 2012;6: 158–170. 10.1038/ismej.2011.75 21716305 PMC3246232

[3] Teschler JK, Zamorano-Sánchez D, Utada AS, Warner CJ, Wong GC, Linington RG, et al Living in the matrix: assembly and control of *Vibrio cholerae* biofilms. Nat Rev Microbiol. 2015;13: 255–268. 10.1038/nrmicro3433 25895940 PMC4437738

[4] Frank DN, Wilson SS, St Amand AL, Pace NR. Culture-independent microbiological analysis of foley urinary catheter biofilms. PloS One. 2009;4: e7811 10.1371/journal.pone.0007811 19907661 PMC2771765

[5] Peleg AY, Hooper DC. Hospital-acquired infections due to gram-negative bacteria. N Engl J Med. 2010;362: 1804–1813. 10.1056/NEJMra0904124 20463340 PMC3107499

[6] Zago CE, Silva S, Sanitá PV, Barbugli PA, Dias CMI, Lordello VB, et al Dynamics of Biofilm Formation and the Interaction between *Candida albicans* and Methicillin-Susceptible (MSSA) and -Resistant *Staphylococcus aureus* (MRSA). PLoS ONE. 2015;10: e0123206 10.1371/journal.pone.0123206 25875834 PMC4395328

[7] Cho I, Blaser MJ. The human microbiome: at the interface of health and disease. Nat Rev Genet. 2012;13: 260–270. 10.1038/nrg3182 22411464 PMC3418802

[8] Oh J, Byrd AL, Deming C, Conlan S, Kong HH, Segre JA, et al Biogeography and individuality shape function in the human skin metagenome. Nature. 2014;514: 59–64. 10.1038/nature13786 25279917 PMC4185404

[9] Peterson J, Garges S, Giovanni M, McInnes P, Wang L, Schloss JA, et al The NIH human microbiome project. Genome Res. 2009;19: 2317–2323. 10.1101/gr.096651.109 19819907 PMC2792171

[10] Milferstedt K, Santa-Catalina G, Godon J-J, Escudié R, Bernet N. Disturbance frequency determines morphology and community development in multi-species biofilm at the landscape scale. 2013; 8:11:e80692 10.1371/journal.pone.0080692 24303024 PMC3841191

[11] Ohsumi T, Takenaka S, Wakamatsu R, Sakaue Y, Narisawa N, Senpuku H, et al Residual Structure of *Streptococcus mutans* Biofilm following Complete Disinfection Favors Secondary Bacterial Adhesion and Biofilm Re-Development. PloS One. 2015;10:1:e0116647 10.1371/journal.pone.0116647 25635770 PMC4312048

[12] Peterson BW, He Y, Ren Y, Zerdoum A, Libera MR, Sharma PK, et al Viscoelasticity of biofilms and their recalcitrance to mechanical and chemical challenges. FEMS Microbiol Rev. 2015;39: 234–245. 10.1093/femsre/fuu008 25725015 PMC4398279

[13] Semenyuk EG, Laning ML, Foley J, Johnston PF, Knight KL, Gerding DN, et al Spore formation and toxin production in *Clostridium difficile* biofilms. PloS One. 2014;9:1: p.e87757 10.1371/journal.pone.0087757 24498186 PMC3907560

[14] Steunou A-S, Bhaya D, Bateson MM, Melendrez MC, Ward DM, Brecht E, et al *In situ* analysis of nitrogen fixation and metabolic switching in unicellular thermophilic cyanobacteria inhabiting hot spring microbial mats. Proc Natl Acad Sci U S A. 2006;103: 2398–2403. 16467157 10.1073/pnas.0507513103 PMC1413695

[15] Ursell T, Chau RMW, Wisen S, Bhaya D, Huang KC. Motility enhancement through surface modification is sufficient for cyanobacterial community organization during phototaxis. 2013;9:9:e1003205 10.1371/journal.pcbi.1003205 24039562 PMC3763999

[16] Wu S, Li X, Gunawardana M, Maguire K, Guerrero-Given D, Schaudinn C, et al Beta-Lactam Antibiotics Stimulate Biofilm Formation in Non-Typeable *Haemophilus influenzae* by Up-Regulating Carbohydrate Metabolism. 2014; 9:7:e99204 10.1371/journal.pone.0099204 25007395 PMC4090067

[17] Mazumdar V, Amar S, Segrè D. Metabolic proximity in the order of colonization of a microbial community. 2013;8:10:e77617 10.1371/journal.pone.0077617 24204896 PMC3813667

[18] Williamson KS, Richards LA, Perez-Osorio AC, Pitts B, McInnerney K, Stewart PS, et al Heterogeneity in *Pseudomonas aeruginosa* biofilms includes expression of ribosome hibernation factors in the antibiotic-tolerant subpopulation and hypoxia-induced stress response in the metabolically active population. J Bacteriol. 2012;194: 2062–2073. 10.1128/JB.00022-12 22343293 PMC3318454

[19] Peters BM, Jabra-Rizk MA, Graeme A, Costerton JW, Shirtliff ME. Polymicrobial interactions: impact on pathogenesis and human disease. Clin Microbiol Rev. 2012;25: 193–213. 10.1128/CMR.00013-11 22232376 PMC3255964

[20] Shade A, Peter H, Allison SD, Baho DL, Berga M, Bürgmann H, et al Fundamentals of microbial community resistance and resilience. 2012 10.3389/fmicb.2012.00417 23267351 PMC3525951

[21] Lindell D, Sullivan MB, Johnson ZI, Tolonen AC, Rohwer F, Chisholm SW. Transfer of photosynthesis genes to and from *Prochlorococcus* viruses. Proc Natl Acad Sci U S A. 2004;101: 11013–11018. 15256601 10.1073/pnas.0401526101 PMC503735

[22] Ogilvie LA, Bowler LD, Caplin J, Dedi C, Diston D, Cheek E, et al Genome signature-based dissection of human gut metagenomes to extract subliminal viral sequences. Nat Commun. 2013;4. 10.1038/ncomms3420 PMC3778543 24036533

[23] Thompson LR, Zeng Q, Kelly L, Huang KH, Singer AU, Stubbe J, et al Phage auxiliary metabolic genes and the redirection of cyanobacterial host carbon metabolism. Proc Natl Acad Sci. 2011;108: E757–E764. 10.1073/pnas.1102164108 21844365 PMC3182688

[24] Breitbart M, Salamon P, Andresen B, Mahaffy JM, Segall AM, Mead D, et al Genomic analysis of uncultured marine viral communities. Proc Natl Acad Sci. 2002;99: 14250–14255. 12384570 10.1073/pnas.202488399 PMC137870

[25] Emerson JB, Thomas BC, Andrade K, Heidelberg KB, Banfield JF. New approaches indicate constant viral diversity despite shifts in assemblage structure in an Australian hypersaline lake. Appl Environ Microbiol. 2013;79: 6755–6764. 10.1128/AEM.01946-13 23995931 PMC3811486

[26] Pride DT, Salzman J, Haynes M, Rohwer F, Davis-Long C, White RA, et al Evidence of a robust resident bacteriophage population revealed through analysis of the human salivary virome. ISME J. 2012;6: 915–926. 10.1038/ismej.2011.169 22158393 PMC3329113

[27] Sullivan MB, Coleman ML, Weigele P, Rohwer F, Chisholm SW. Three *Prochlorococcus* cyanophage genomes: signature features and ecological interpretations. PLoS Biol. 2005;3: e144 15828858 10.1371/journal.pbio.0030144 PMC1079782

[28] Bhaya D, Grossman AR, Steunou A-S, Khuri N, Cohan FM, Hamamura N, et al Population level functional diversity in a microbial community revealed by comparative genomic and metagenomic analyses. ISME J. 2007;1: 703–713. 18059494 10.1038/ismej.2007.46

[29] Gomez-Garcia MR, Davison M, Blain-Hartnung M, Grossman AR, Bhaya D. Alternative pathways for phosphonate metabolism in thermophilic cyanobacteria from microbial mats. ISME J. 2011;5: 141–149. 10.1038/ismej.2010.96 20631809 PMC3105666

[30] Jensen SI, Steunou A-S, Bhaya D, Kühl M, Grossman AR. In situ dynamics of O2, pH and cyanobacterial transcripts associated with CCM, photosynthesis and detoxification of ROS. ISME J. 2011;5: 317–328. 10.1038/ismej.2010.131 20740024 PMC3105686

[31] Klatt CG, Inskeep WP, Herrgard MJ, Jay ZJ, Rusch DB, Tringe SG, et al Community structure and function of high-temperature chlorophototrophic microbial mats inhabiting diverse geothermal environments. Front Microbiol. 2013;4:106 10.3389/fmicb.2013.00106 23761787 PMC3669762

[32] Heidelberg JF, Nelson WC, Schoenfeld T, Bhaya D. Germ warfare in a microbial mat community: CRISPRs provide insights into the co-evolution of host and viral genomes. PLoS One. 2009;4:4169–4169. 10.1371/journal.pone.0004169 PMC2612747 19132092

[33] Schoenfeld T, Patterson M, Richardson PM, Wommack KE, Young M, Mead D. Assembly of viral metagenomes from Yellowstone hot springs. Appl Environ Microbiol. 2008;74: 4164–4174. 10.1128/AEM.02598-07 18441115 PMC2446518

[34] Labrie SJ, Samson JE, Moineau S. Bacteriophage resistance mechanisms. Nat Rev Microbiol. 2010;8: 317–327. 10.1038/nrmicro2315 20348932

[35] Barrangou R, Fremaux C, Deveau H, Richards M, Boyaval P, Moineau S, et al CRISPR provides acquired resistance against viruses in prokaryotes. Science. 2007;315: 1709–1712. 17379808 10.1126/science.1138140

[36] Bhaya D, Davison M, Barrangou R. CRISPR-Cas systems in bacteria and archaea: versatile small RNAs for adaptive defense and regulation. Annu Rev Genet. 2011;45: 273–297. 10.1146/annurev-genet-110410-132430 22060043

[37] Paez-Espino D, Morovic W, Sun CL, Thomas BC, Ueda K, Stahl B, et al Strong bias in the bacterial CRISPR elements that confer immunity to phage. Nat Commun. 2013;4: 1430 10.1038/ncomms2440 23385575

[38] Deveau H, Barrangou R, Garneau JE, Labonté J, Fremaux C, Boyaval P, et al Phage response to CRISPR-encoded resistance in *Streptococcus thermophilus*. J Bacteriol. 2008;190: 1390–1400. 18065545 10.1128/JB.01412-07 PMC2238228

[39] Andersson AF, Banfield JF. Virus population dynamics and acquired virus resistance in natural microbial communities. Science. 2008;320: 1047–1050. 10.1126/science.1157358 18497291

[40] Held NL, Herrera A, Cadillo-Quiroz H, Whitaker RJ. CRISPR associated diversity within a population of *Sulfolobus islandicus*. PLoS One. 2010 9 28;5:9:e12988 10.1371/journal.pone.0012988 20927396 PMC2946923

[41] Skennerton CT, Imelfort M, Tyson GW. Crass: identification and reconstruction of CRISPR from unassembled metagenomic data. Nucleic Acids Res. 2013;183. 10.1093/nar/gkt183 PMC3664793 23511966

[42] Treangen TJ, Koren S, Sommer DD, Liu B, Astrovskaya I, Ondov B, et al MetAMOS: a modular and open source metagenomic assembly and analysis pipeline. Genome Biol. 2013;14 14:1:R2 10.1186/gb-2013-14-1-r2 23320958 PMC4053804

[43] Sundquist A, Bigdeli S, Jalili R, Druzin ML, Waller S, Pullen KM, et al Bacterial flora-typing with targeted, chip-based Pyrosequencing. BMC Microbiol. 2007;7: 108 18047683 10.1186/1471-2180-7-108 PMC2244631

[44] Parks DH, MacDonald NJ, Beiko RG. Classifying short genomic fragments from novel lineages using composition and homology. BMC Bioinformatics. 2011;12: 328 10.1186/1471-2105-12-328 21827705 PMC3173459

[45] Buckley DH, Graber JR, Schmidt TM. Phylogenetic analysis of nonthermophilic members of the kingdom Crenarchaeota and their diversity and abundance in soils. Appl Environ Microbiol. 1998;64: 4333–4339. 9797286 10.1128/aem.64.11.4333-4339.1998 PMC106648

[46] Schleper C, Jurgens G, Jonuscheit M. Genomic studies of uncultivated archaea. Nat Rev Microbiol. 2005;3: 479–488. 15931166 10.1038/nrmicro1159

[47] Boomer SM, Noll KL, Geesey GG, Dutton BE. Formation of multilayered photosynthetic biofilms in an alkaline thermal spring in Yellowstone National Park, Wyoming. Appl Environ Microbiol. 2009;75: 2464–2475. 10.1128/AEM.01802-08 19218404 PMC2675224

[48] Klatt CG, Bryant DA, Ward DM. Comparative genomics provides evidence for the 3-hydroxypropionate autotrophic pathway in filamentous anoxygenic phototrophic bacteria and in hot spring microbial mats. Environ Microbiol. 2007;9: 2067–2078. 17635550 10.1111/j.1462-2920.2007.01323.x

[49] Miyake K, Abe K, Ferri S, Nakajima M, Nakamura M, Yoshida W, et al A green-light inducible lytic system for cyanobacterial cells. Biotechnol Biofuels. 2014;7:56 10.1186/1754-6834-7-56 24713090 PMC4021604

[50] Eddy SR. Accelerated profile HMM searches. PLoS Comput Biol. 2011;7:e1002195 10.1371/journal.pcbi.1002195 22039361 PMC3197634

[51] Namiki T, Hachiya T, Tanaka H, Sakakibara Y. MetaVelvet: an extension of Velvet assembler to de novo metagenome assembly from short sequence reads. Nucleic Acids Res. 2012;40: e155–e155. 10.1093/nar/gks678 22821567 PMC3488206

[52] Teeling H, Glöckner FO. Current opportunities and challenges in microbial metagenome analysis—a bioinformatic perspective. Brief Bioinform. 2012; bbs039. 10.1093/bib/bbs039 PMC3504927 22966151

[53] Yilmaz S, Allgaier M, Hugenholtz P. Multiple displacement amplification compromises quantitative analysis of metagenomes. Nat Methods. 2010;7: 943–944. 10.1038/nmeth1210-943 21116242

[54] Bankevich A, Nurk S, Antipov D, Gurevich AA, Dvorkin M, Kulikov AS, et al SPAdes: a new genome assembly algorithm and its applications to single-cell sequencing. J Comput Biol. 2012;19: 455–477. 10.1089/cmb.2012.0021 22506599 PMC3342519

[55] Meyer F, Paarmann D, D’Souza M, Olson R, Glass EM, Kubal M, et al The metagenomics RAST server–a public resource for the automatic phylogenetic and functional analysis of metagenomes. BMC Bioinformatics. 2008;9: 386 10.1186/1471-2105-9-386 18803844 PMC2563014

[56] Wilmes P, Andersson AF, Lefsrud MG, Wexler M, Shah M, Zhang B, et al Community proteogenomics highlights microbial strain-variant protein expression within activated sludge performing enhanced biological phosphorus removal. ISME J. 2008;2: 853–864. 10.1038/ismej.2008.38 18449217

[57] Dick GJ, Andersson AF, Baker BJ, Simmons SL, Thomas BC, Yelton AP, et al Community-wide analysis of microbial genome sequence signatures. Genome Biol. 2009;10: R85 10.1186/gb-2009-10-8-r85 19698104 PMC2745766

[58] Strous M, Kraft B, Bisdorf R, Tegetmeyer HE. The binning of metagenomic contigs for microbial physiology of mixed cultures. Front Microbiol. 2012;3. 10.3389/fmicb.2012.00410 PMC3514610 23227024

[59] Ultsch A, Mörchen F. ESOM-Maps: tools for clustering, visualization, and classification with Emergent SOM. 2005;

[60] Rombel IT, Sykes KF, Rayner S, Johnston SA. ORF-FINDER: a vector for high-throughput gene identification. Gene. 2002;282: 33–41. 11814675 10.1016/s0378-1119(01)00819-8

[61] Zdobnov EM, Apweiler R. InterProScan–an integration platform for the signature-recognition methods in InterPro. Bioinformatics. 2001;17: 847–848. 11590104 10.1093/bioinformatics/17.9.847

[62] Emerson D, Field EK, Chertkov O, Davenport KW, Goodwin L, Munk C, et al Comparative genomics of freshwater Fe-oxidizing bacteria: implications for physiology, ecology, and systematics. Front Microbiol. 2013;4. 10.3389/fmicb.2013.00254 PMC3770913 24062729

[63] Mizuno CM, Rodriguez-Valera F, Garcia-Heredia I, Martin-Cuadrado A-B, Ghai R. Reconstruction of novel cyanobacterial siphovirus genomes from Mediterranean metagenomic fosmids. Appl Environ Microbiol. 2013;79: 688–695. 10.1128/AEM.02742-12 23160125 PMC3553755

[64] Lopes A, Tavares P, Petit M-A, Guérois R, Zinn-Justin S. Automated classification of tailed bacteriophages according to their neck organization. BMC Genomics. 2014;15: 1027 10.1186/1471-2164-15-1027 25428721 PMC4362835

[65] Waterhouse AM, Procter JB, Martin DM, Clamp M, Barton GJ. Jalview Version 2—a multiple sequence alignment editor and analysis workbench. Bioinformatics. 2009;25: 1189–1191. 10.1093/bioinformatics/btp033 19151095 PMC2672624

[66] Dereeper A, Guignon V, Blanc G, Audic S, Buffet S, Chevenet F, et al Phylogeny. fr: robust phylogenetic analysis for the non-specialist. Nucleic Acids Res. 2008;36: W465–W469. 10.1093/nar/gkn180 18424797 PMC2447785

[67] Young R. Phage lysis: Three steps, three choices, one outcome. J Microbiol. 2014;52: 243–258. 10.1007/s12275-014-4087-z 24585055 PMC4012431

[68] Oliveira H, Melo LD, Santos SB, Nóbrega FL, Ferreira EC, Cerca N, et al Molecular aspects and comparative genomics of bacteriophage endolysins. J Virol. 2013;87: 4558–4570. 10.1128/JVI.03277-12 23408602 PMC3624390

[69] Rice P, Longden I, Bleasby A. EMBOSS: the European molecular biology open software suite. Trends Genet. 2000;16: 276–277. 10827456 10.1016/s0168-9525(00)02024-2

[70] Schuster-Böckler B, Schultz J, Rahmann S. HMM Logos for visualization of protein families. BMC Bioinformatics. 2004;5: 7 14736340 10.1186/1471-2105-5-7 PMC341448

[71] Howe AC, Jansson JK, Malfatti SA, Tringe SG, Tiedje JM, Brown CT. Tackling soil diversity with the assembly of large, complex metagenomes. Proc Natl Acad Sci. 2014;111: 4904–4909. 10.1073/pnas.1402564111 24632729 PMC3977251

[72] Loman NJ, Constantinidou C, Chan JZ, Halachev M, Sergeant M, Penn CW, et al High-throughput bacterial genome sequencing: an embarrassment of choice, a world of opportunity. Nat Rev Microbiol. 2012;10: 599–606. 10.1038/nrmicro2850 22864262

[73] Simpson JT, Pop M. The Theory and Practice of Genome Sequence Assembly. Annu Rev Genomics Hum Genet. 2015; 16:153–72 10.1146/annurev-genom-090314-050032 25939056

[74] Rosen MJ, Davison M, Bhaya D, Fisher DS. Fine-scale diversity and extensive recombination in a quasisexual bacterial population occupying a broad niche. Science. 2015;348: 1019–1023. 10.1126/science.aaa4456 26023139

[75] Dutilh BE, Cassman N, McNair K, Sanchez SE, Silva GGZ, Boling L, et al A highly abundant bacteriophage discovered in the unknown sequences of human faecal metagenomes. Nat Commun. 2014;5:4498 10.1038/ncomms5498 25058116 PMC4111155

[76] Hurwitz BL, Sullivan MB. The Pacific Ocean Virome (POV): A Marine Viral Metagenomic Dataset and Associated Protein Clusters for Quantitative Viral Ecology. PLoS ONE. 2013;8: e57355 10.1371/journal.pone.0057355 23468974 PMC3585363

[77] Roux S, Hallam SJ, Woyke T, Sullivan MB. Viral dark matter and virus-host interactions resolved from publicly available microbial genomes. eLife. 2015;22:4 10.7554/eLife.08490 PMC4533152 26200428

[78] Clokie MR, Millard AD, Letarov AV, Heaphy S. Phages in nature. Bacteriophage. 2011;1:31–45. 21687533 10.4161/bact.1.1.14942 PMC3109452

[79] Edwards RA, Rohwer F. Viral metagenomics. Nat Rev Microbiol. 2005;3:504–510. 15886693 10.1038/nrmicro1163

[80] Sakowski EG, Munsell EV, Hyatt M, Kress W, Williamson SJ, Nasko DJ, et al Ribonucleotide reductases reveal novel viral diversity and predict biological and ecological features of unknown marine viruses. Proc Natl Acad Sci. 2014;111:15786–15791. 10.1073/pnas.1401322111 25313075 PMC4226130

[81] Adriaenssens EM, Cowan DA. Using signature genes as tools to assess environmental viral ecology and diversity. Appl Environ Microbiol. 2014;80: 4470–4480. 24837394 10.1128/AEM.00878-14 PMC4148782

[82] Kimura S, Sako Y, Yoshida T. Rapid gene diversification of *Microcystis* cyanophages revealed by long-and short-term genetic analysis of the tail sheath gene in a natural pond. Appl Environ Microbiol. 2013; AEM. 03751–12. 10.1128/AEM.03751-12 PMC3623194 23417006

[83] Sullivan MB. Viromes, Not Gene Markers, for Studying Double-Stranded DNA Virus Communities. J Virol. 2015;89: 2459–2461 10.1128/JVI.03289-14 25540374 PMC4325738

[84] Pope PB, Totsika M, de Carcer DA, Schembri MA, Morrison M. Muramidases found in the foregut microbiome of the Tammar wallaby can direct cell aggregation and biofilm formation. ISME J. 2011;5:341–350. 10.1038/ismej.2010.116 20668486 PMC3105685

[85] Rodríguez-Rubio L, Gutiérrez D, Donovan DM, Martínez B, Rodríguez A, García P. Phage lytic proteins: biotechnological applications beyond clinical antimicrobials. Crit Rev Biotechnol. 2015; 1–11. 10.3109/07388551.2014.993587 25603721

[86] Mao J, Schmelcher M, Harty WJ, Foster-Frey J, Donovan DM. Chimeric Ply187 endolysin kills *Staphylococcus aureus* more effectively than the parental enzyme. FEMS Microbiol Lett. 2013;342: 30–36. 10.1111/1574-6968.12104 23413880 PMC3690576

[87] Schmelcher M, Donovan DM, Loessner MJ. Bacteriophage endolysins as novel antimicrobials. Future Microbiol. 2012;7: 1147–1171. 10.2217/fmb.12.97 23030422 PMC3563964

[88] Gervasi T, Horn N, Wegmann U, Dugo G, Narbad A, Mayer MJ. Expression and delivery of an endolysin to combat *Clostridium perfringens*. Appl Microbiol Biotechnol. 2014;98: 2495–2505. 10.1007/s00253-013-5128-y 23942878 PMC3936119

[89] Mayer MJ, Gasson MJ, Narbad A. Genomic sequence of bacteriophage ATCC 8074-B1 and activity of its endolysin and engineered variants against *Clostridium sporogenes*. Appl Environ Microbiol. 2012;78: 3685–3692. 10.1128/AEM.07884-11 22427494 PMC3346354

[90] Briers Y, Walmagh M, Van Puyenbroeck V, Cornelissen A, Cenens W, Aertsen A, et al Engineered endolysin-based “artilysins” to combat multidrug-resistant gram-negative pathogens. MBio. 2014;5: e01379–14. 10.1128/mBio.01379-14 24987094 PMC4161244

[91] Sundquist A, Bigdeli S, Jalili R, Druzin ML, Waller S, Pullen KM, et al Bacterial flora-typing with targeted, chip-based Pyrosequencing. BMC Microbiol. 2007;7: 108 18047683 10.1186/1471-2180-7-108 PMC2244631

[92] Grissa I, Vergnaud G, Pourcel C. The CRISPRdb database and tools to display CRISPRs and to generate dictionaries of spacers and repeats. BMC Bioinformatics. 2007;8: 172 17521438 10.1186/1471-2105-8-172 PMC1892036

[93] Altschul SF, Gish W, Miller W, Myers EW, Lipman DJ. Basic local alignment search tool. J Mol Biol. 1990;215: 403–410. 2231712 10.1016/S0022-2836(05)80360-2

[94] Grissa I, Vergnaud G, Pourcel C. CRISPRFinder: a web tool to identify clustered regularly interspaced short palindromic repeats. Nucleic Acids Res. 2007;35: W52–W57. 17537822 10.1093/nar/gkm360 PMC1933234

[95] Johnson M, Zaretskaya I, Raytselis Y, Merezhuk Y, McGinnis S, Madden TL. NCBI BLAST: a better web interface. Nucleic Acids Res. 2008;36: W5–W9. 10.1093/nar/gkn201 18440982 PMC2447716

[96] Edgar RC. MUSCLE: multiple sequence alignment with high accuracy and high throughput. Nucleic Acids Res. 2004;32: 1792–1797. 15034147 10.1093/nar/gkh340 PMC390337

[97] Markowitz VM, Chen I-MA, Chu K, Szeto E, Palaniappan K, Pillay M, et al IMG/M 4 version of the integrated metagenome comparative analysis system. Nucleic Acids Res. 2014;42: D568–D573. 10.1093/nar/gkt919 24136997 PMC3964948

