# Supplementary figures and images for: Diversity in a Polymicrobial Community Revealed by Analysis of Viromes, Endolysins and CRISPR Spacers
Source: PLoS One. 2016 Sep 9;11(9):e0160574. doi: 10.1371/journal.pone.0160574 (PMC5017753; doi:10.1371/journal.pone.0160574)

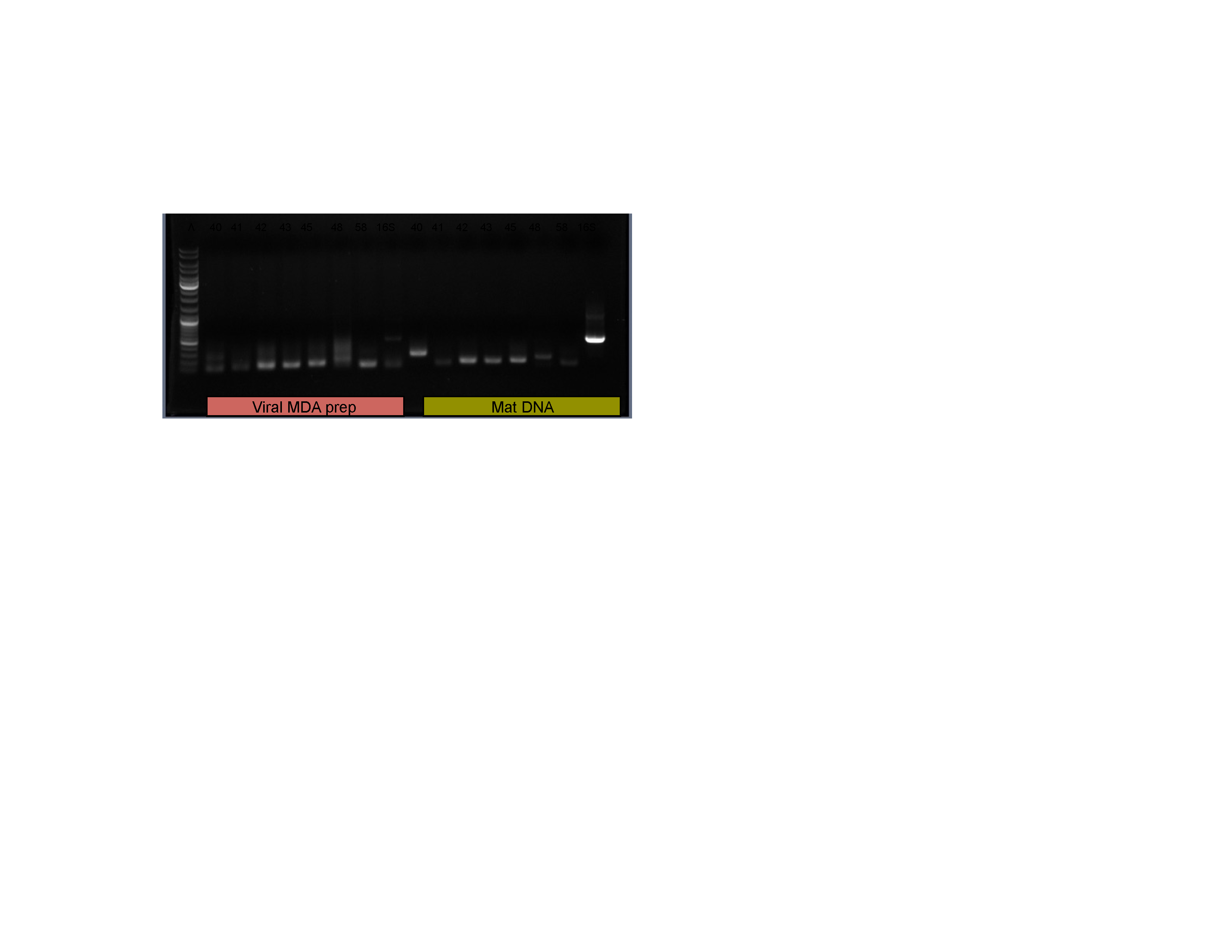

Supplement: S1 Fig — Viral reads present in OS-V-03 and BP-V-03 were used to generate viral specific primers (wells 40–58). General bacterial 16S RNA primers V1for and V3rev [91] were used to amplify a 460bp fragment. An intense16S band is observed in Mat DNA, while the amount of 16S present in the viral MDA prep is very faint. (TIFF) [file pone.0160574.s001.tiff]

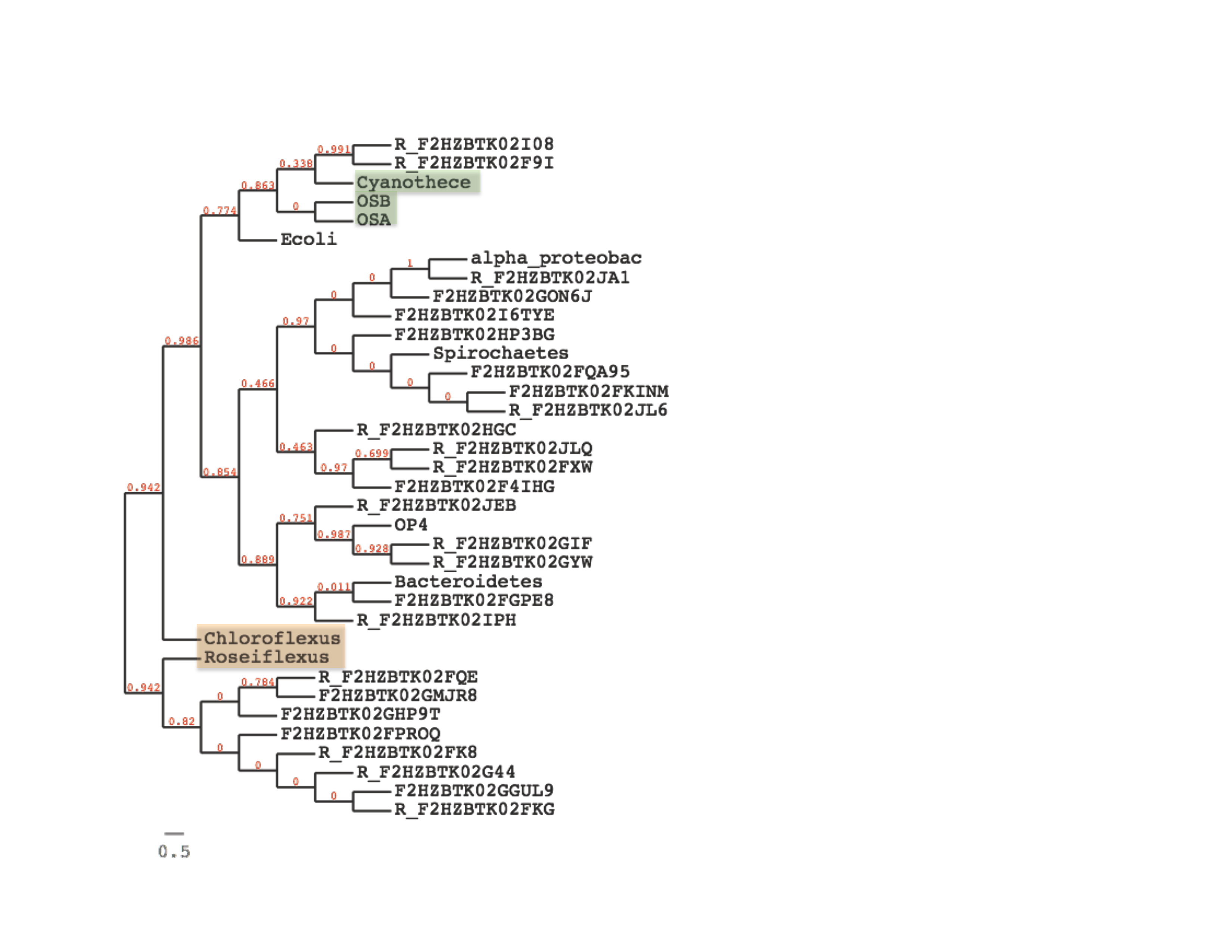

Supplement: S2 Fig — Phylogeny of twenty-six identified 16S viral reads with known organisms. Cyanobacteria are marked in green, while Chloroflexii in orange. (TIFF) [file pone.0160574.s002.tiff]

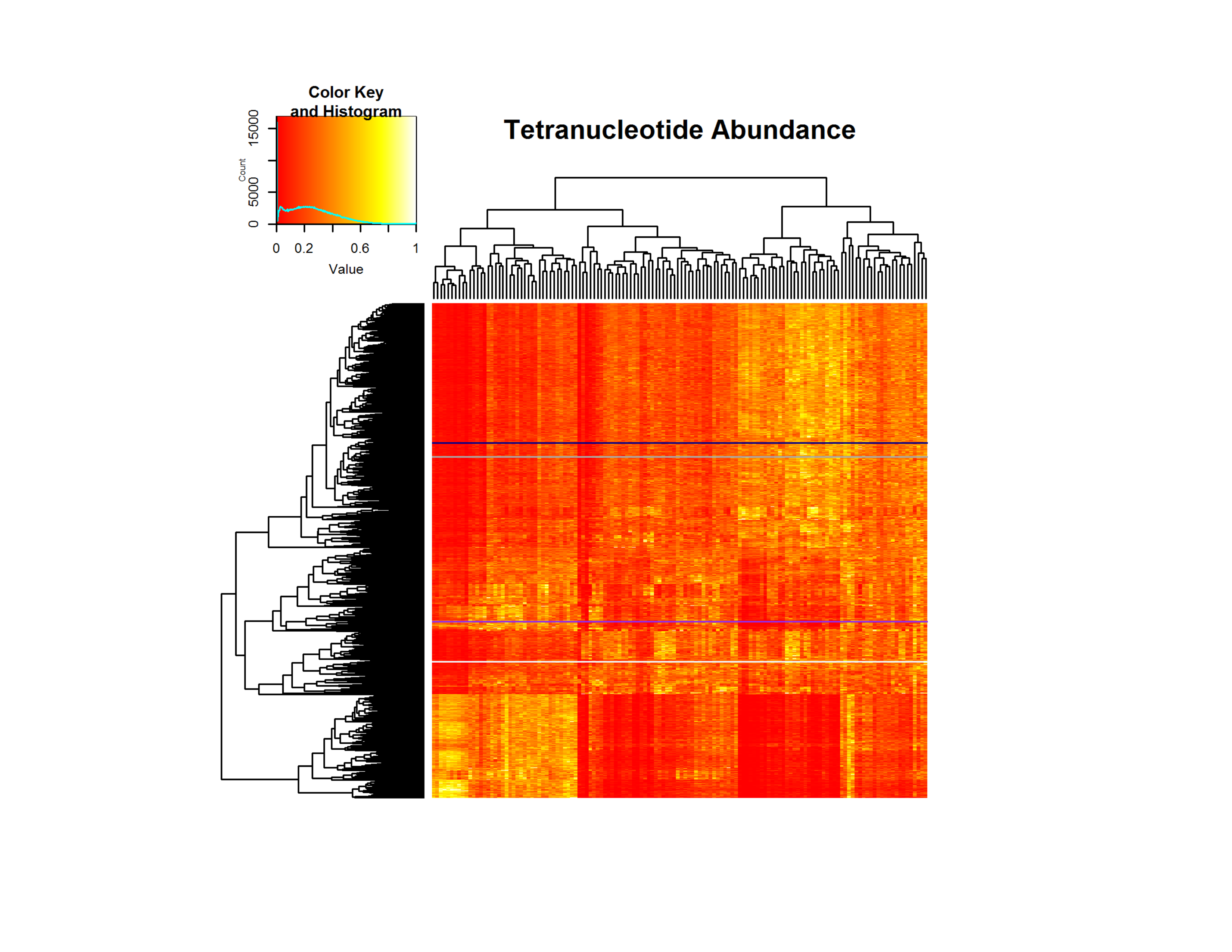

Supplement: S3 Fig — Contigs greater than 1Kb were pipelined through custom scripts by Dick et al [57] to calculate tetranucleotide frequency. (TIFF) [file pone.0160574.s003.tiff]

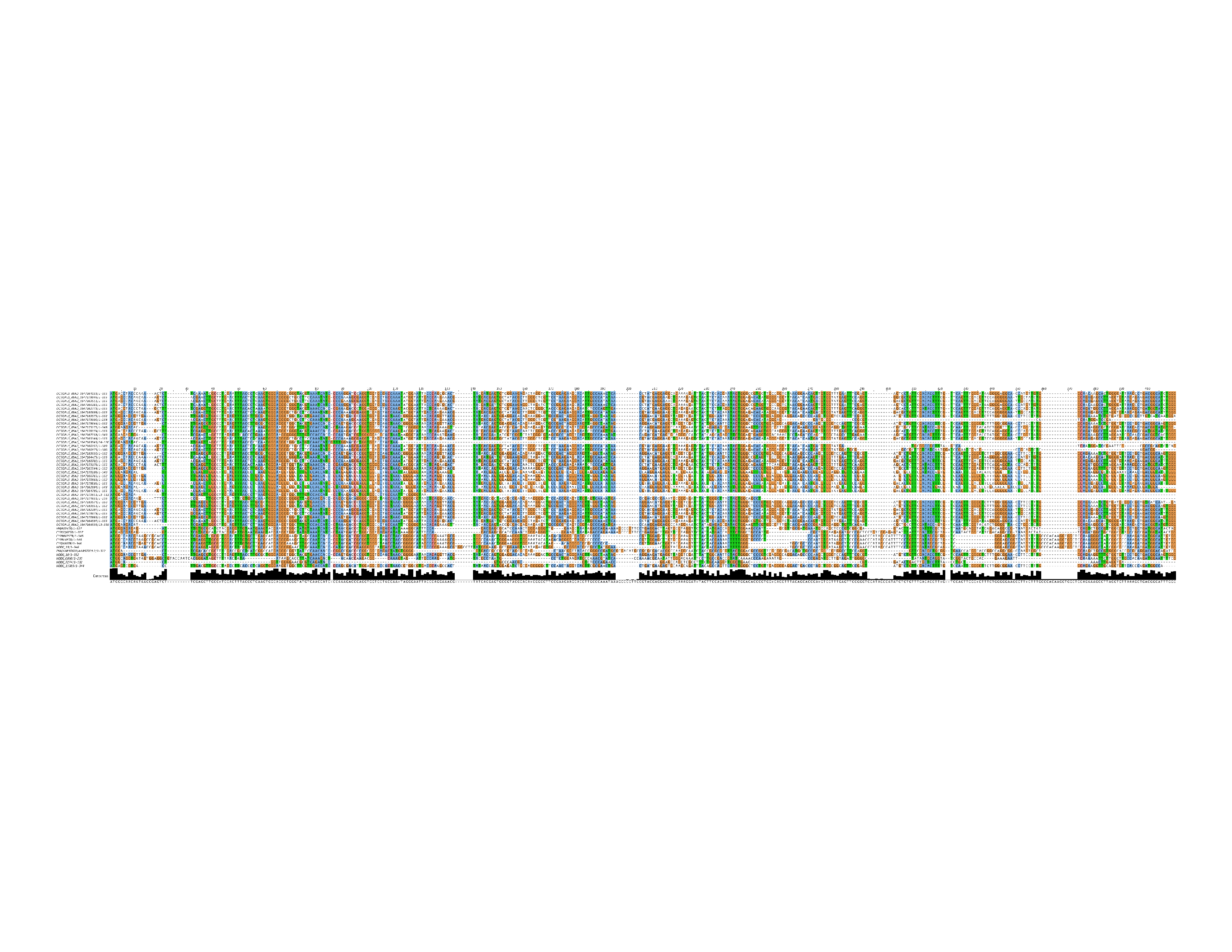

Supplement: S4 Fig — (TIFF) [file pone.0160574.s004.tiff]
